# Provision of Hospital Accommodation

**Published:** 1921-02-12

**Authors:** 


### PROVISION OF HOSPITAL ACCOMMODATION.

By AN INFIRMARY MEDICAL SUPERINTENDENT.

### II. The Poor-Law Infirmary.

BESIDES the financial differences already mentioned, the Poor-law infirmary differs from all other British hospitals in the legal relations between it and its patients, in its functions, and in its organisation. The patient in a Poor-law infirmary is not there as an object of charity or because his disease is infectious or his conduct differs from the normal; he is there by right of sickness, of poverty, and of settlement in the parish. Possessed of these three credentials, he has a legal right of admission to the infirmary. He cannot be refused because the infirmary is full or because he is dirty or verminous, incontinent, incurable, or of unpleasing habits. He cannot be told to wait his turn on a list, or required to bring a number of things for his personal use with him. On the other hand, the infirmary has a legal claim upon him. Since the poverty that entitles him to admission is not absolute destitution, but is the inability to provide for himself the medical treatment necessary for the relief of his sickness, he can be called upon to contribute, so far as he is able, to the cost of his maintenance.

### A WIDE AND EVER-OPEN DOOR.

This legal right to admission gives to the infirmary its peculiar characteristics. It is a hospital for every kind and degree of sickness. Patients old and young, men and women, with trivial complaints, acutely and seriously ill, needing surgical operations, suffering from chronic and incurable diseases—all must be admitted and kept until they die, or until they are restored to a wage-earning condition or to a state in which their friends are willing to take them home again. The patient cannot be discharged on any other grounds. The functions of a Poor-law hospital include those of an acute and chronic hospital, a home for the dying, a colony for epileptics and the feeble-minded, a lock hospital, a maternity hospital, a children's hospital, a pre-maternity and children's care centre, a sanatorium for early and advanced tuberculosis, an infectious hospital for chicken-pox, measles, whooping-cough, erysipelas, and puerperal fever, a reception-house for cases of mental disease before certification, a home for chronic and harmless insanity, a cleansing centre for scabies and the verminous, a convalescent home, a neurological hospital, and every other kind of special hospital. The poor suffering from disease for which charity and the municipal authorities have provided insufficient or no accommodation find the doors of the infirmary open to them; the police, with a verminous, filthy, or drunken victim of a street accident, or some wretched outcast collapsed from starvation and neglect, in their hands, know that, though the voluntary hospital may refuse him, the Poor-law infirmary will take him in without formality.

### THE WORKHOUSE BLIGHT.

These are the demands upon the Poor-law infirmary. What provisions have the Guardians made to meet them? It is a question not easy to answer succinctly, because the provision made varies widely in different parishes. The first institutions provided under the Poor-law were the workhouses, intended for the temporary relief of those without work, and deliberately organised so that existence within them should be less pleasant than that of the poorest self-supporting person outside them. The Poor-law infirmary has grown out of these workhouses, and, unfortunately, the dread of making them too attractive has hung over them from their inception like a blight, a blight from which they have only in recent years begun to escape. The workhouses, from their beginning, had to provide for sick inmates, and they did so by setting aside wards within the building for their accommodation. Later, in many parishes this accommodation was found to be unsuitable and insufficient, and then special buildings were erected within the workhouse grounds. The medical care of the inmates was placed in the hands of the workhouse medical offices—in the smaller houses a part-time and non-resident, in the larger a whole-time and resident, official. In either case, as a rule, the general administration of the wards, including the control of the nursing staff and of the patients and helpers, was in the hands of the workhouse master and matron.

In London, and later in some of the larger provincial towns, the separate infirmary came into being. Though the administration of these infirmaries is entirely separate from that of the workhouses, there are usually important links between the two institutions. As a rule, the two buildings adjoin, the washing of the infirmary is done in the workhouse laundry, the engineering, carpentering, and similar services in the infirmary are carried out by the workhouse staff, inmates of the workhouse are employed in the domestic work of the infirmary, patients are transferred freely from the one institution to the other, and the medical care of the inmates of the workhouse is in the charge of the infirmary medical staff. In some cases it is in the workhouse that one finds the lying-in, the prematernity, the lunatic, and epileptic wards, the nursery, and wards for the infirm, crippled, and some classes of chronic patients.

The whole of the administration of a separate infirmary is in the hands of the medical superintendent. He is responsible for the admission and discharge of patients, for their treatment and dieting, for the keeping of records and statistics, for the control of the nursing and domestic staffs, for the training of the probationers, and for the economical

The first article appeared on Jan. 29, p. 407.

**Provision of Hospital Accommodation**—(*continued*).

and efficient working of the institution. He is assisted by one or more medical officers, by a matron, and by a steward; but all matters of difficulty, all changes in routine, are referred to him for settlement and approval. He works under the control of an Infirmary Committee appointed by the Board of Guardians. To this Committee all important changes in routine and all matters involving new expenditure must be referred for decision.

Handicaps and Possibilities.

In a Poor-law infirmary the first things that strike any one accustomed to the working of a voluntary hospital are the paucity of the medical and nursing staffs, the absence of a specialist medical staff, the meagreness of the equipment for diagnosis and treatment when the variety and complexity of the diseases treated is taken into account, and the absence of an out-patient department. It is true things are not quite so bad as they were a few years ago. The hours of work of the nursing and domestic staffs have been shortened, the number of nurses and, to a less extent, of the doctors has been increased; in the larger infirmaries an *x*-ray equipment and facilities for pathological and bacteriological work have been provided, and two or three specialists have been appointed. In the Paddington Infirmary a more ambitious scheme has been started. This infirmary has entered into an arrangement with St. Mary's Hospital by which the members of the hospital staff are available for consultation by the staff of the infirmary, and the students of the hospital are able to take part in the clinical work of the infirmary. Notwithstanding these reforms, the problem of dealing efficiently with all the patients in the infirmary has not yet been seriously tackled. But in London a beginning has been made. Here the Metropolitan Asylums Board has undertaken work which points the way to the solution. This Board, made up of representatives of the London Boards of Guardians and of co-opted members, was started to deal with cases of fever and of chronic insanity. It has extended its work, and now has many special hospitals, dealing chiefly with the diseases of children. The relief would be greater if there were less delay in getting the transfers effected.

The possible usefulness of the Poor-law infirmary is greatly curtailed by the objection to entering them as patients shown by many for whom hospital treatment is necessary. The infirmary ought to be a truly democratic institution, giving to each according to his needs, and exacting from him according to his means. Unfortunately, most Boards of Guardians slurred over the giving and stressed the exacting. Feeling their responsibilities as spenders of the ratepayers' money, they tried to restrict admission to the infirmary to those for whom no other relief by charity, or otherwise, could be obtained, and starved the remedial services they provided. In consequence the public came to look upon the infirmary as the last resort, meant only for the very poor and friendless. The kindlier and more generous policy of recent years is lessening this prejudice, but far more radical changes are necessary before one can expect it finally to disappear.

Empty Beds: An Explanation.

Dr. Addison has said recently that there are 30,000 empty beds in the Poor-law infirmaries—nearly one-third of their total accommodation. The practical value of the statement is stultified by the fact that we do not know at what time of the year the census was taken. In London in the winter all the infirmaries are full, or nearly full; in summer a quarter to a third of their beds are empty. These empty beds might, and ought to be, utilised, but the great difficulty is to find beds for the "peak load" of winter sickness. What evidence there is points to the fact that a useful but not very large number could be found in the infirmaries.

This review of the Poor-law infirmary is imperfect, because details and exceptional conditions have been ignored; but one may deduce from it and the preceding article the broad outlines of the position.

